# Key pathways regulated by *HoxA9,10,11*/*HoxD9,10,11* during limb development

**DOI:** 10.1186/s12861-015-0078-5

**Published:** 2015-07-19

**Authors:** Anna M. Raines, Bliss Magella, Mike Adam, S. Steven Potter

**Affiliations:** Division of Developmental Biology, Cincinnati Children’s Medical Center, 3333 Burnet Ave., Cincinnati, OH 45229 USA

**Keywords:** Hox genes, Limb development, RNA-Seq, Zone of polarizing activity, Apical ectodermal ridge, Zeugopod, Endochondral bone development, Sonic hedgehog, Fgf8, Lef1

## Abstract

**Background:**

The 39 mammalian Hox genes show problematic patterns of functional overlap. In order to more fully define the developmental roles of Hox genes it is necessary to remove multiple combinations of paralogous and flanking genes. In addition, the downstream molecular pathways regulated by Hox genes during limb development remain incompletely delineated.

**Results:**

In this report we examine limb development in mice with frameshift mutations in six Hox genes*, Hoxa9,10,11* and *Hoxd9,10,11*. The mice were made with a novel recombineering method that allows the simultaneous targeting of frameshift mutations into multiple flanking genes. The *Hoxa9,10,11*^*−/−*^/*Hoxd9,10,11*^*−/−*^ mutant mice show a reduced ulna and radius that is more severe than seen in *Hoxa11*^−/−^/*Hoxd11*^−/−^ mice, indicating a minor role for the flanking *Hox9,10* genes in zeugopod development, as well as their primary function in stylopod development. The mutant mice also show severe reduction of *Shh* expression in the zone of polarizing activity, and decreased *Fgf8* expression in the apical ectodermal ridge, thereby better defining the roles of these specific Hox genes in the regulation of critical signaling centers during limb development. Importantly, we also used laser capture microdissection coupled with RNA-Seq to characterize the gene expression programs in wild type and mutant limbs. Resting, proliferative and hypertrophic compartments of E15.5 forelimb zeugopods were examined. The results provide an RNA-Seq characterization of the progression of gene expression patterns during normal endochondral bone formation. In addition of the Hox mutants showed strongly altered expression of *Pknox2*, *Zfp467*, *Gdf5*, *Bmpr1b*, *Dkk3*, *Igf1*, *Hand2*, *Shox2*, *Runx3*, *Bmp7* and *Lef1*, all of which have been previously shown to play important roles in bone formation.

**Conclusions:**

The recombineering based frameshift mutation of the six flanking and paralogous *Hoxa9,10,11* and *Hoxd9,10,11* genes provides a resource for the analysis of their overlapping functions. Analysis of the *Hoxa9,10,11*^*−/−*^*/Hoxd9,10,11*^−/−^ mutant limbs confirms and extends the results of previous studies using mice with Hox mutations in single paralogous groups or with entire Hox cluster deletions. The RNA-Seq analysis of specific compartments of the normal and mutant limbs defines the multiple key perturbed pathways downstream of these Hox genes.

**Electronic supplementary material:**

The online version of this article (doi:10.1186/s12861-015-0078-5) contains supplementary material, which is available to authorized users.

## Background

Hox genes encode transcription factors that are among the key regulators of development. Mutations of *Drosophila* Hox genes often result in dramatic homeotic transformations of body parts, with the morphology of one segment altered to resemble that of another. For example, the *Antennapedia* mutation, resulting in ectopic expression, causes imaginal discs that would normally form antennae to instead make legs that now protrude from the head [1]. Mutation of mammalian Hox genes, however, generally results in milder phenotypes, which is attributed to their greater number and functional redundancy.

The 39 mammalian Hox genes are arranged in four clusters (A-D). On the basis of sequence similarities and positions within the clusters the Hox genes can be divided into 13 paralogous groups (1–13) [2, 3]. In many developing systems the Hox genes show nested domains of overlapping expression. This led to the suggestion that combinatorial codes of Hox expression could drive segment identity determination. Consistent with this, Hox mutations can cause partial or complete homeotic transformations in the development of the hindbrain, reproductive tracts, and axial skeleton. For example, mutation of *Hoxb1* causes rhombomere (r) 4 of the hindbrain to assume the character of r2 [4], and ectopic expression of *Hoxa1* or *Hoxb1* results in the transformation of r2 into r4 [5]. Mutation of *Hoxa9,10,11*/*Hoxd9,10,11* genes gives partial homeotic transformations of uterus to oviduct and vas deferens to seminiferous tubules [6, 7]. Further, mutational analysis shows that most of the Hox genes of paralogous groups 3–13 play roles in defining segment identities of the axial skeleton [8].

Hox mutations do not, however, produce homeotic transformations in the developing mammalian limbs. Mutations of single Hox genes result in very subtle developmental defects in the limbs. For example, homozygous mutation of *Hoxa11* gives slightly misshapen ulna and radius, and fusion of the triangular and pisiform carpal bones [9]. Similarly, mutation of the paralog *Hoxd11* gives modest defects in the distal ends of the ulna and radius [10]. Combined mutation of both *Hoxa11* and *Hoxd11*, however, gives a striking reduction in size of the ulna and radius (zeugopod), illustrating the important functional redundancy of these paralogs [11]. Coupled with the known expression patterns of Hox genes in the developing limbs this led to a model with *Hox9* and *Hox10* paralog genes responsible for patterning the stylopod (humerus); *Hox11* genes, the zeugopod; and *Hox12* and *Hox13* genes, the wrist and autopod (paw) skeletal elements [11]. This model has generally held true, with confirming examples including dramatic truncation of hindlimb stylopod (femur) in *Hoxa10/Hoxc10/Hoxd10* mutants [12]; of hindlimb zeugopod (tibia and fibula) in *Hoxa11/Hoxc11/Hoxd11* mutants [12]; and complete loss of all autopod elements in *Hoxa13/Hoxd13* mutants [13]. It is also clear from these studies that genes of the *HoxA* and *HoxD* clusters are primarily responsible for forelimb patterning, while *HoxC* cluster function contributes to hindlimb patterning.

The functional overlap of Hox genes is not, however, restricted to paralogous groups. There is considerable evidence showing that Hox genes flanking each other within a cluster are also functionally redundant. The amino acid sequences of the DNA binding homeodomains encoded by flanking Hox genes often approach the degree of similarity seen among paralogs. Further, flanking Hox genes sometimes show more similar expression patterns than paralogs, consistent with shared regional enhancers [14]. *Hoxa10*/*Hoxa11* trans-heterozygotes show synergistic phenotypes reflecting functional redundancy of these two flanking Hox genes [15]. Non-paralogous mutations in *Hoxa10/Hoxd11* result in forelimb defects not seen in either single mutant [16]. In addition, homeobox swap experiments have shown that the homeodomains of flanking Hox genes are often, but not always, functionally equivalent [17, 18]. In sum, it is clear that in order to fully reveal Hox functions it is necessary to mutate both paralogous and flanking Hox genes.

To achieve this end one strategy is to create LoxP mediated deletions that include multiple flanking Hox genes [19]. This approach has indeed been very informative, for example in defining novel kidney malformations resulting when several flanking HoxD genes are removed [20–22]. Nevertheless, the results are challenging to interpret because the deletion of a region of a Hox cluster removes shared enhancers, which results in misexpression of the remaining Hox genes.

An extreme example of this strategy is to use Cre/LoxP to remove an entire Hox cluster [23–25]. The results show that removal of a single Hox cluster can give a surprisingly mild phenotype. For example, HoxC cluster deletion mice survive to birth with only minor malformations apparent [24]. Seeming paradoxical, the removal of the entire HoxC cluster results in a phenotype milder in many respects than the mutation of the single *Hoxc9* gene [24]. At least in some cases there appears to be noncoding RNA mediated cross regulation between Hox clusters, such that removal of one cluster results in compensatory up-regulation of others [26]. Entire Hox cluster deletions therefore are useful, but coarse and imperfect tools for the study of Hox gene functional relationships.

In this report we used a different strategy. A novel version of recombineering allows the simultaneous introduction of frameshift mutations into multiple flanking genes [7]. In this manner we disrupted the coding regions of paralog group 9,10,11 genes of both the HoxA and HoxD clusters. With this approach the intergenic noncoding RNAs and enhancers remain intact, thereby promoting maintenance of normal Hox cluster cross regulation and normal expression of Hox genes that were not mutated. In this study we define the limb malformations that result when *Hoxa9,10,11* and *Hoxd9,10,11* are mutated.

Limbs achieve their final conformation in two stages. The first stage (from approximately E9 – E12) is characterized by formation and outgrowth of the limb bud, which determines the number of mesenchymal progenitor cells available for skeletal element condensations [27]. Limb bud outgrowth is controlled primarily by fibroblast growth factors (FGFs) emanating from the apical ectodermal ridge (AER) [28–30]. Full expression of FGFs in the AER is dependent on signals from the mesenchyme, including *Sonic hedgehog* (*Shh*) in the zone of polarizing activity (ZPA) [31]. In the second stage of limb development (from E12.5 onward), chondrocytes proliferate to extend the length of limb condensations, and then differentiate into a hypertrophic state; finally forming ossified long bones through the process of endochondral ossification [32]. Hox genes contribute to the patterning of specific limb elements via involvement in both of these stages of limb development. Deletion of the entire *HoxA* and *HoxD* clusters results in a near complete loss of forelimb skeletal elements, with significantly retarded outgrowth of the limb bud due to loss of *Shh* signaling in the ZPA and severe disruption of *Fgf8* signaling in the AER [23], as well as reduced *Grem1* expression [33].

Defects in chondrocyte differentiation leading to failure of long bone outgrowth have been observed in the zeugopods of *Hoxa11*^*−/−*^*/Hoxd11*^*−/−*^ mutant mice [34, 35]. The mesenchymal condensations giving rise to forelimb zeugopod elements initiate normally in *Hoxa11*^*−/−*^*/Hoxd11*^*−/−*^ mutants, although these condensations are smaller in mutants vs. WT mice as early as E12.5 [34]. The *Hoxa11*^*−/−*^*/Hoxd11*^*−/−*^ mutant zeugopod also initiates type II collagen expression, suggesting that there is not a defect in chondrogenesis. Analysis at later stages however, shows that chondrocyte differentiation into the hypertrophic state is severely delayed in the mutant ulna and radius [34, 35]. There is eventually some hypertrophic differentiation of *Hoxa11*^*−/−*^*/Hoxd11*^*−/−*^ mutant chondrocytes in the zeugopod, resulting in small centers of ossification in these significantly shortened bones. Interestingly, E18.5 mice with three mutant alleles of *Hoxa11* and *Hoxd11* displayed only a slight shortening of zeugopod bones but were found to have shortened growth plates and delayed ossification postnatally [34].

A key challenge in the study of Hox genes is to define their overlapping functions. To reveal redundant functions it is necessary to remove increasing layers of related Hox genes. To define their molecular mechanisms of action it is necessary to analyze the perturbed gene expression programs in Hox mutants.

In this report we examine limb development in mice with frameshift mutations in six Hox genes*, Hoxa9,10,11* and *Hoxd9,10,11*. Gross skeletal abnormalities are defined, showing a zeugopod reduction in *Hoxa9,10,11*^*−/−*^/*Hoxd9,10,11*^*−/−*^ mice that is significantly more severe than observed for *Hoxa11*^*−/−*^/*Hoxd11*^*−/−*^ mice. This indicates a minor role of the flanking *Hoxa9,10* and *Hoxd9,10* genes in zeugopod development, as well as their primary role in stylopod development. In addition, the relative contributions of the 9,10,11 paralog genes of the A and D clusters are defined, and perturbed expression of Shh and Fgf8, markers of the ZPA and AER respectively, characterized. Of particular interest, we also used laser capture microdissection (LCM) coupled with RNA-Seq to define the gene expression patterns in the zeugopods of the wild type and *Hoxa9,10,11*^*−/−*^/*Hoxd9,10,11*^*−/−*^ mutant mice. The results provide a global view of the changing gene expression programs in the normal resting, proliferative, and hypertrophic zones of the wild type developing bone. These results also define key perturbed pathways in Hox mutant limbs. In particular, we observe altered expression of a number of genes known to regulate chondrocyte or osteoblast differentiation, including *Pknox2*, *Zfp467*, *Gdf5*, *Bmpr1b*, *Dkk3*, *Igf1*, *Hand2*, *Shox2*, *Runx3*, *Bmp7* and *Lef1*. The results provide a global view of the molecular pathways and biological processes downstream of the *Hoxa9,10,11*/*Hoxd9,10,11* genes in the developing limb.

## Methods

### Mice

A modified recombineering strategy was used to generate BAC targeting constructs over 100 Kb in length that facilitated the simultaneous frameshift mutation of *Hoxa9,10,11* and *Hoxd9,10,11* genes, as previously described [7]. For each of the six mutated Hox genes the coding region of the canonical first exon was disrupted through a LoxP insertion, coupled with a small flanking deletion, resulting in a frameshift mutation [7]. All experiments were carried out with humane protocols (protocol number 2D12115) approved by the Institutional Animal Care and Use Committee.

### Skeletal staining and histological analysis

Skeletons of E18.5 embryos were stained with alcian blue (cartilage) and alizarin red following standard protocols [36]. For histological analysis, embryonic limbs were fixed in 4 % paraformaldehyde, and then processed for paraffin embedding. Hematoxylin and eosin and Safranin-Weigert staining were performed following standard protocols. For immunofluorescence, slides were probed with an antibody to Sox9 (Millipore, AB5535, 1:500), Six2 (Proteintech, 11562-1-AP, 1:100), Lef1 (Cell Signaling, 2230P, 1:100), Gas1 (R&D, AF2644, 1:100), or Runx3 (Novus, NB100-91276, 1:200). Staining of wild type and mutant sections was carried out on the same slide, and visualization carried out with the same microscope/photography settings. Antibody specificity was shown using control tissues with known expression patterns.

### Whole-mount in situ hybridization

Whole mount *in situ* hybridization was performed following standard procedures using previously described antisense riboprobes for *Shh* [37] and *Fgf8* [38]. *Hoxd12* and *Hoxd13* antisense riboprobes were generated via *in vitro* transcription of PCR products generated with the following primers: *Hoxd12*: T7 promoter *AACTAATACGACTCACTATAGGG*CCGGTTTTCAACGTGTTCTT, Sp6 promoter A*ACGATTTAGGTGACACTATAG*GATAGGTGAGGCTGGAGCAG, *Hoxd13*: T7 promoter *AACTAATACGACTCACTATAGGG*CCAGGCCAGTATGAGGAAAA, Sp6 promoter A*ACGATTTAGGTGACACTATAG*CCCCCAAATGAATTTCAGAA.

### Laser capture microdissection and RNA isolation

Forelimbs were dissected from E15.5 embryos, placed in ice-cold PBS, and washed 3× in OCT before embedding in OCT and flash frozen. Limbs were then sectioned at 10 μM thickness onto membrane slides (Applied Biosystems) and stored at −80 °C. LCM of resting, proliferative, and hypertrophic zeugopod chondrocytes from WT (*n* = 3) and undifferentiated chondrocytes from *aadd* mutants (*n* = 3) was performed with the Arcturus Veritas system. The cells isolated were predominantly chondrocytes, but with some perichondrial contribution. Captured cells were immediately transferred into 50 μL of lysis buffer (0.05 % SDS, 5 mM Tris pH 7), vortexed and frozen at −80 °C until RNA isolation with Qiagen RNeasy Mini kit. RNA quality and concentration were assessed using Agilent RNA 6000 Pico Chip on the Agilent 2100 Bioanalyzer.

### RNA-Seq

Total RNAs (20 ng/sample) from the chondrocyte populations isolated via laser capture microdissection were prepared for RNA-Seq using the NOvation® RNA-Seq System V2 (NuGen). Data was analyzed in GeneSpring 12.6.1 as previously described [39]. Bam files were generated using mouse build mm10. Data was filtered on read quality metrics, including removal of reads aligning to more than one position in the genome, and duplicate reads. Data was filtered on expression level, requiring at least 5 NRPKM in three samples. Analyses were carried out with both ANOVA (all samples) and moderated T-test (two way comparisons), requiring *P* < 0.05. The *aadd* versus wild type comparison was also carried out with Audic Claverie Test pooled RNA-Seq reads (*P* ≤ 0.05), which detected more differences than the moderated T-test. Various fold change criteria were used as described in the text. Gene Ontology analysis was carried out with ToppGene (https://toppgene.cchmc.org) [40]. Data was deposited in the GEO database [GSE66679].

## Results and discussion

### Gross analysis of *Hoxa9,10,11*/*Hoxd9,10,11* mutant limbs

We used a novel recombineering strategy to introduce small deletion/frameshift mutations into the first exons of each of the *Hoxa9*, *Hoxa10*, *Hoxa11*, *Hoxd9*, *Hoxd10* and *Hoxd11* genes as previously described [7]. By using BAC targeting constructs over 100 Kb in length it was possible to simultaneously target three flanking Hox genes. In this report mice with homozygous mutation in all three flanking *Hoxa9,10,11* genes are referred to as *aa, Hoxd9,10,11*^−/−^*mice are dd,* and mice double homozygous mutant for both *Hoxa9,10,11* and *Hoxd9,10,11* are *aadd*, while wild type mice are *AADD*.

**Fig. 1:**
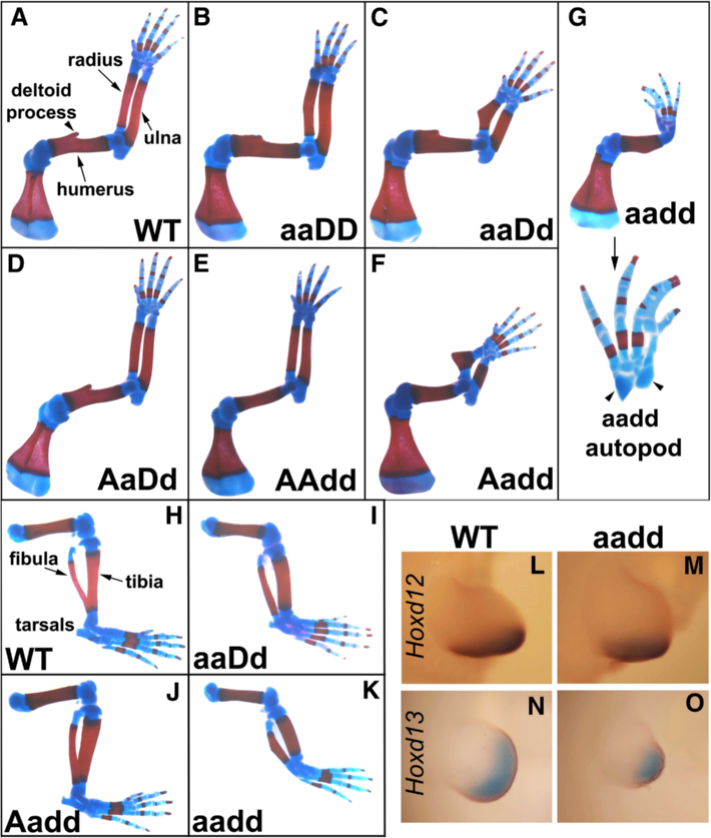
Limb malformations in mice with mutations of *Hoxa9,10,11* and *Hoxd9,10,11*. **a**–**k**: Alcian blue (cartilage) and Alizarin red [52] staining of E18.5 limbs. **a**–**g** Forelimb **a**: wild type, note size of radius, ulna, humerus, and deltoid process. **b**: *Hoxa9,10,11* ^−/−^, thickening of the radius and ulna. **c**: *Hoxa9,10,11* ^−/−^;*Hoxd9,10,11* ^+/−^, thickening and shortening of the radius and ulna, slight outgrowth of the radius. **d**: *Hoxa9,10,11* ^+/−^;*Hoxd9,10,11* ^+/−^, comparable to wild type in size and shape. **e**: *Hoxd9,10,11* ^−/−^, thin humerus, and absent deltoid process. **f**: *Hoxa9,10,11* ^+/−^;*Hoxd9,10,11* ^−/−^, absent deltoid process, severe shortening of the radius and ulna, large outgrowth on the radius. **g**: *Hoxa9,10,11* ^−/−^;*Hoxd9,10,11* ^−/−^ absent deltoid process, near absence of the radius and ulna, syndactyly of digits 2 and 3. **h**–**k** Hindlimb **h**: Wild type, note size and separation of tibia, fibula, and tarsals. **i**: *Hoxa9,10,11* ^−/−^;*Hoxd9,10,11* ^−/−^, Complete distal separation and thickening of the tibia and fibula. **j**: *Hoxa9,10,11* ^+/−^; *Hoxd9,10,11* ^−/−^, thickening of the tibia and fibula. **k**: *Hoxa9,10,11* ^−/−^;*Hoxd9,10,11* ^−/−^, Shortening, thickening, and separation of the tibia and fibula, absence of tarsal. **l**–**o**: *In situ* hybridization of E11.5 forelimb bud. **l**–**m** Hoxd12 *in situ* probe. **l**: wild type. **m**: *Hoxa9,10,11* ^−/−^;*Hoxd9,10,11* ^−/−^. **n**–**o** *Hoxd1*3 *in situ* probe. **n**: wild type. **o**: *Hoxa9,10,11* ^−/−^;*Hoxd9,10,11* ^−/−^. Note similar expression levels in mutants

Double homozygous mutation of the two paralogous genes *Hoxa11* and *Hoxd11* results in severe shortening of the radius and ulna [11, 34]. But even *Hox11* triple mutants, with all paralog group 11 genes mutated (*Hoxa11*, *Hoxc11* and *Hoxd11*), retain small centers of ulna and radius ossification [12]. It was therefore interesting that the E18.5 *aadd* mutant skeletons showed near complete loss of forelimb zeugopod elements, with only very small cartilage remnants of ulna and radius (Fig. 1g vs. WT in Fig. 1a). This indicates a supporting role for flanking *Hox9* and *Hox10* paralog group genes in patterning of the zeugopod, in addition to their primary function in stylopod development. These results confirm and extend previous observations. *Hox10* triple mutants show modest shortening of the forelimb zeugopod as well as the predicted more severe shortening of the stylopod, [12]. Further, mice null for the entire *HoxA* cluster in the limb show defects in the zeugopod that are more severe than those observed in *Hoxa11* mutants, suggesting the participation of flanking genes on the *HoxA* cluster [23].

The *aadd* forelimb also displayed a shortened humerus, which is predicted by the mutation of 9 and 10 paralog genes (Fig. 1g). Notably, however, the *aadd* stylopod mutant phenotypes were milder than might have been expected, given that both paralog 9 and 10 genes of the Hox A and D clusters were mutated. The *Hoxa9*^−/−^/*Hoxd9*^−/−^ mutant humerus shows a mild shortening and reduced deltoid tuberosity [41]. Similar, the *Hoxa10*^−/−^/*Hoxd10*^−/−^ mutants also show stylopod shortening and in this case complete loss of the deltoid tuberosity [12]. The *aadd* humerus shows shortening that appears, based on published images, somewhat more severe than either the *Hoxa9*^−/−^/*Hoxd9*^−/−^ or *Hoxa10*^−/−^/*Hoxd10*^−/−^ mutants, as well as loss of the deltoid tuberosity, but not the level of synergistic severity that one might expect.

As expected, with the known restricted role of HoxC genes in hindlimb development, the hindlimb malformations in *aadd* mice were much milder than for forelimb, with a zeugopod that was somewhat shortened and misshapen (Fig. 1k vs. WT in Fig. 1h).

We also found a number of autopod defects in the *aadd* mutant forelimb; including fusion of digits 2 and 3, and loss or fusion of several wrist bones (Fig. 1g). Although fusion of carpal bones and shortening of phalanges have been reported in mice with targeted mutations in *Hoxa11* [9] and *Hoxd11* [10], respectively, major autopod defects typically occur only upon mutation or misexpression of Hox12 or Hox13 genes. Therefore, we performed whole mount *in situ* hybridization for *Hoxd12* and *Hoxd13* on E11.5 embryos, looking for possible secondary changes in their expression patterns. We found no detectable difference in the intensity of *Hoxd12* or *Hoxd13* expression in *aadd* mutant limb buds (Fig. 1m, o) compared to WT (Fig. 1l, n). Nevertheless, the mutant limbs were somewhat altered in shape, raising the possibility that the relative domains of *Hoxd12* or *Hoxd13* expression were changed. Of interest, a previous report using a series of mutant mice with five nested HoxD gene deletions concluded that Hox paralog groups 10, and 11 contribute to autopod development [42].

We also found evidence of functional overlap among flanking Hox genes through analysis of mutant limbs with additional mutant allele combinations. Although newborn mouse skeletons missing three alleles of *Hoxa11/d11* have near normal length forelimb zeugopod bones [34], we found a significantly shortened zeugopod and misshapen radius in both *aaDd* and *Aadd* (9-allele mutant) E18.5 mice (Fig. 1c, f). This further shows that the paralog group 9 and 10 Hox genes contribute significantly, along with group 11, to zeugopod development. *Aadd* mutant hindlimbs were normal (Fig. 1j), and only minor thickening of the tibia and fibula were observed in the *aaDd* mutant (Fig. 1i).

The *Aadd* and *aaDd* mutant phenotypes indicate that the *Hoxd9,10,11* genes play a more important role in forelimb patterning than the *Hoxa9,10,11* genes. First, the ulna and radius of *Aadd* mutants were consistently shorter than in *aaDd* mutants (compare Fig. 1f with 1c). Second, the humerus of the *Aadd* mutant forelimb was significantly shortened and lacked the characteristic deltoid process normally found on this bone (Fig. 1f). In addition, we observed a thinned or shortened humerus missing the deltoid process in 30 % of *AAdd* mutants (data not shown and Fig. 1e). The *aaDD* mutant forelimbs displayed some minor abnormalities including thickening of zeugopod bones and wrist bone fusions (Fig. 1b). Interestingly, we found no abnormalities in forelimbs of *AaDd* mutants (Fig. 1d), consistent with some specific HoxA and HoxD functions, in addition to their strongly overlapping roles.

### ZPA and AER signaling is disrupted in *Hoxa9,10,11/Hoxd9,10,11* mutant forelimbs

**Fig. 2:**
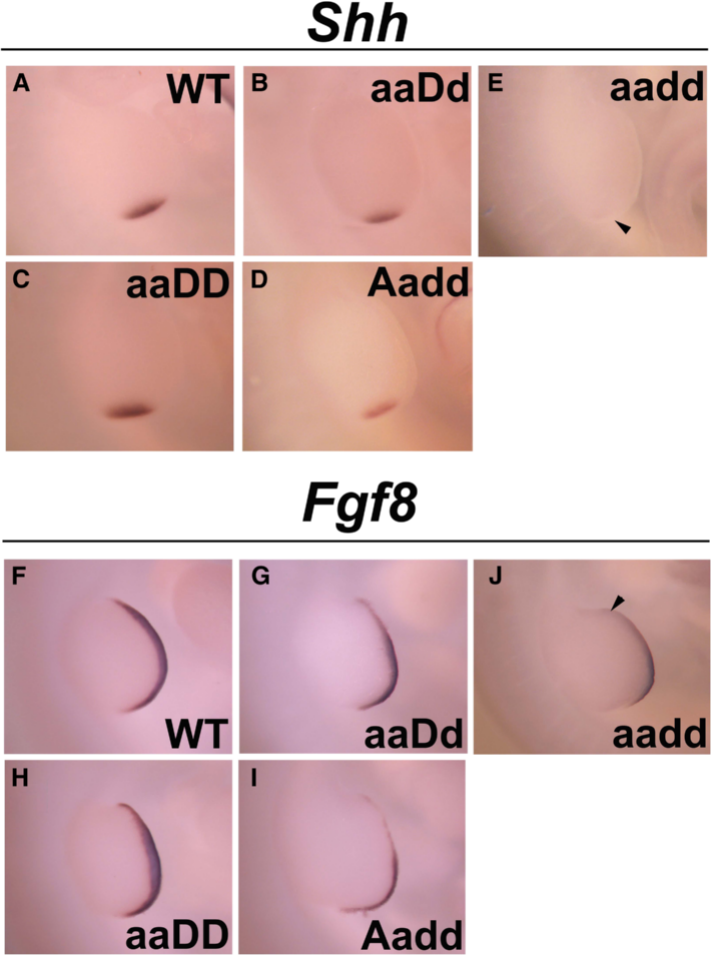
Altered *Shh* and *Fgf8* expression in E10.5 *Hoxa9,10,11*/*Hoxd9,10,11* mutants. **a**–**e**: Shh expression in the ZPA. **a**: Wild type. **b**: *Hoxa9,10,11* ^−/−^;*Hoxd9,10,11* ^+/−^, reduced expression level. **c**: *Hoxa9,10,11* ^−/−^, normal expression. **d**: *Hoxa9,10,11* ^+/−^;*Hoxd9,10,11* ^−/−^, decreased expression. **e**: *Hoxa9,10,11* ^−/−^;*Hoxd9,10,11* ^−/−^, nearly absent expression, arrowhead pointing to ZPA. **f**–**j**: *Fgf8* expression in the AER. **f**: Wild type. **g**: *Hoxa9,10,11* ^−/−^;*Hoxd9,10,11* ^+/−^, reduced anterior expression. **h**: *Hoxa9,10,11* ^−/−^, normal expression. **i**: *Hoxa9,10,11* ^+/−^;*Hoxd9,10,11* ^−/−^ reduced and patchy expression. **j**: *Hoxa9,10,11* ^−/−^;*Hoxd9,10,11* ^−/−^, Decreased expression, arrowhead pointing to anterior edge of the AER, where expression was most severely reduced

To determine the effect of *Hoxa9,10,11/Hoxd9,10,11* mutations on factors governing limb bud outgrowth at early stages, we performed whole mount *in situ* hybridization for key signaling molecules in the ZPA (*Shh*) and the AER (*Fgf8*) in E10.5 embryos. Similar to a previous study looking at *Shh* signal in forelimb buds of mutants with increasing depletion of entire *HoxA/D* cluster genes [23], we found a dose—response relationship between the number of intact *Hoxa/d9,10,11* genes and the strength of *Shh* signal in the ZPA at E10.5 (Fig. 2). The *aadd* mutants showed very weak *Shh* expression (Fig. 2e), while both *aAdd* and *aaDd*, displayed somewhat reduced *Shh* expression domains (Fig. 2b, d) compared to WT (Fig. 2a). It is interesting that such reduced SHH expression as observed in *aadd* mutants still supports limb development to the degree observed.

As *Shh* signaling is necessary for proper expression of FGFs in the AER [43], we next looked at *Fgf8* expression in *Hox9,10,11* mutant E10.5 limb buds. Unlike the WT, which showed strong, uniform *Fgf8* expression along the length of the AER (Fig. 2f), *aadd* mutants displayed decreased patchy expression with a faint anterior domain (Fig. 2j). Interestingly, even though there was a clear difference in strength of *Shh* signal in *aadd* vs. *Aadd* and *aaDd* mutants, we did not observe significant difference in *Fgf8* expression among these mutants. Both *aaDd* (Fig. 2g) and *Aadd* mutants (Fig. 2i) showed the same diminished anterior AER expression of *Fgf8* seen in *aadd* mutants. In *aaDD* mutants, which have near normal length forelimb skeletal elements, *Fgf8* expression appeared normal (Fig. 2h).

There is an interesting cross-regulatory loop between SHH and Hox genes. *Shh* mutation perturbs Hox expression [31] and Hox genes are also upstream of *Shh* expression. Hoxd10*,* and Hoxd1*3* proteins bind directly to the *Shh* long-range enhancer to regulate *Shh* expression [44]. A separate study showed that Hoxd12 and Hoxd13 activate *Shh* expression in the ZPA [45]. The strong reduction of *Shh* expression in the *aadd* ZPA demonstrates the dominant role of the Hox9,10,11 genes in *Shh* regulation. The observed remaining minimal expression of *Shh* in *aadd* mutants is likely due, at least in part, to the continued presence of Hoxd12 and Hoxd13. And the reduced *Shh* expression in the *aadd* mutants could result in subtle changes in *Hoxd12* and/or *Hoxd13* expression not readily detected by our *in situ* hybridizations that are then responsible for the observed autopod defects.

### Zeugopod elements initiate in *aadd* mutants but the resulting chondrocytes fail to differentiate

**Fig. 3:**
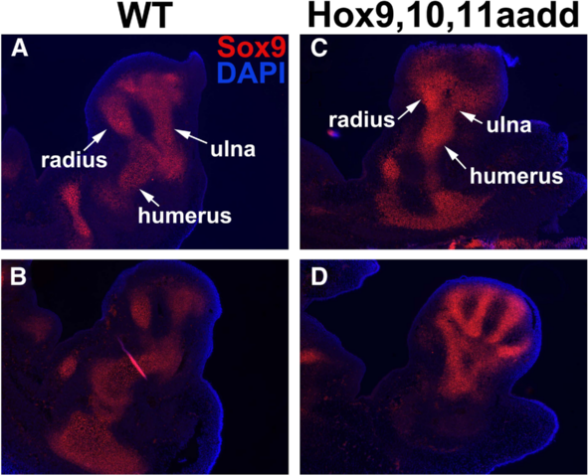
Reduced ulna and radius in *Hoxa9,10,11* ^*−/−*^;*Hoxd9,10,11* ^*−/−*^ mutants at E12.5. **a**–**b** wild type, note radius and ulna size. **c**–**d** *Hoxa9,10,11* ^−/−^;*Hoxd9,10,11* ^−/−^, smaller ulna and radius, with the ulna more severely reduced. Two sections of each are shown to give a more complete view of the malformations. Cartilage is visualized with Sox9 immunostain (*red*). Cell nuclei are visualized with DAPI stain (*blue*)

We next investigated whether the mesenchymal condensations of the zeugopod initiate properly in *aadd* mutants. Immunostaining for Sox9 in sections of E12.5 forelimb buds showed clear condensations of proliferating chondrocytes for ulna, radius, and humerus in WT embryos (Fig. 3a, b). The *aadd* mutant limb buds also showed a clear humerus condensation, which was then bifurcated into two small elements, the ulna and radius, before further branching into the digits of the autopod (Fig. 3c, d). Although present at E12.5, the zeugopod condensations were already clearly smaller in the mutant compared to WT, with the ulna most severely reduced in size.

**Fig. 4:**
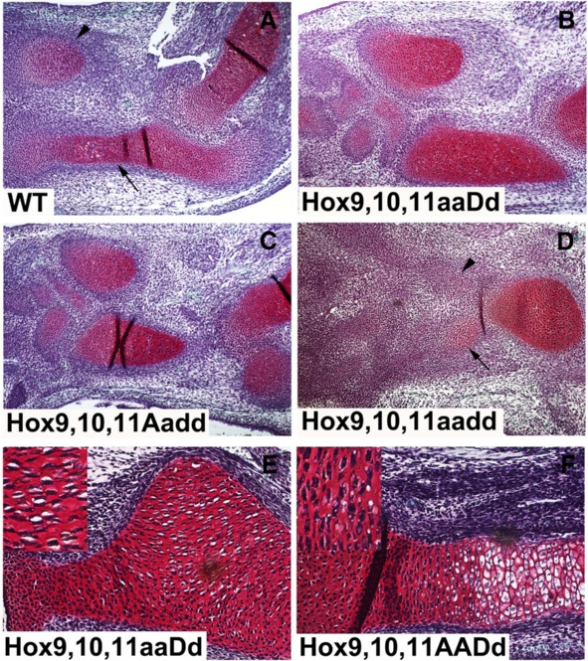
Safranin-Weigert staining of Hox mutant limbs. Arrowhead: radius, Arrow: ulna. **a**–**d**, E14.5. **a**: Wild type, note resting chondrocytes near ends, the proliferative zone more medial, and the hypertrophic compartment, with white spaces. **b**: *Hoxa9,10,11* ^−/−^;*Hoxd9,10,11* ^+/−^, shorter and thicker radius and ulna. **c**: *Hoxa9,10,11* ^+/−^;*Hoxd9,10,11* ^−/−^, shorter and thicker radius and ulna. **d**: *Hoxa9,10,11* ^−/−^;*Hoxd9,10,11* ^−/−^, nearly absent radius and ulna, with only resting chondrocyte morphology. **e** and **f**, E15.5. **e**: *Hoxa9,10,11* ^−/−^;*Hoxd9,10,11* ^+/−^, cells present in outgrowth of radius are rotated 90° (insert). **f**: *Hoxd9,10,11* ^+/^-, normal cell orientation and differentiation compartments (insert)

Analysis of limbs at later stages showed that the chondrocytes in the initial zeugopod condensations in *aadd* forelimbs failed to progress normally in development. The WT E14.5 forelimb showed strong Safranin-Weigert staining in the ulna and radius with obvious morphological differences between resting chondrocytes at the ends of the developing long bones, the more medial proliferating chondrocytes and the hypertrophic chondrocytes located toward the center (Fig. 4a). In contrast, the *aadd* mutants showed very little Safranin-Weigert staining of zeugopod chondrocytes at E14.5 (Fig. 4d). The *aadd* mutant zeugopod chondrocytes appeared histologically to most closely resemble the resting phase of the wild type. The *aadd* mutant stylopod displayed strong staining although this element was clearly small and misshapen compared to the WT (Fig. 4d).

Safranin-Weigert staining of E14.5 *aaDd* (Fig. 4b) and *Aadd* (Fig. 4c) limbs showed a more normal zeugopod although clearly stunted compared to WT. The radius was also misshapen, displaying a characteristic kink in both *aaDd* and *Aadd* mutants. Interestingly, Safranin-Weigert staining at E15.5 revealed a 90° rotation of the columnar pre-hypertrophic chondrocytes in the *aaDd* mutant compared to WT (Fig. 4e vs. 4f). This rotation was also observed in the *Aadd* mutant radius, and suggests that these Hox genes can affect the orientation of chondrocyte cell division.

### RNA-Seq profiling of chondrocytes in WT and *aadd* mutant zeugopods

LCM was used to obtain compartment specific samples from the resting, proliferating, and hypertrophic chondrocyte regions of WT E15.5 zeugopod. Due to the histologic homogeneity and small size of *aadd* mutant zeugopods the entire chondrocyte population was taken as a single sample. Isolated samples were primarily chondrocytes, but included some flanking perichondrial contribution. This approach promotes discovery of gene expression differences in the perichondrium, where Hox gene is strongest [46], as well as the forming chondrocytes, where the resulting downstream phenotypic consequences of Hox mutation are most pronounced. It is important to note that most of the gene expression difference detected likely reflect downstream events in chondrocytes and not direct Hox targets within the perichondrium. RNA-Seq was performed on three independent sets of WT and *aadd* compartments.

**Fig. 5:**
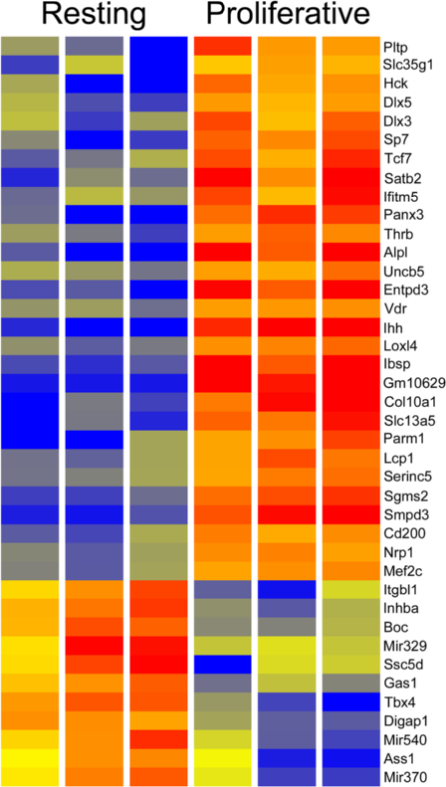
Heatmap comparing wild type resting and proliferative compartments. The 40 genes with the greatest fold change (≥5) are shown. This provides an RNA-Seq analysis of the changing gene expression programs as cells transition from the resting to the proliferative compartments. Red reflects strong, blue represents weak, and yellow indicates intermediate expression

**Fig. 6:**
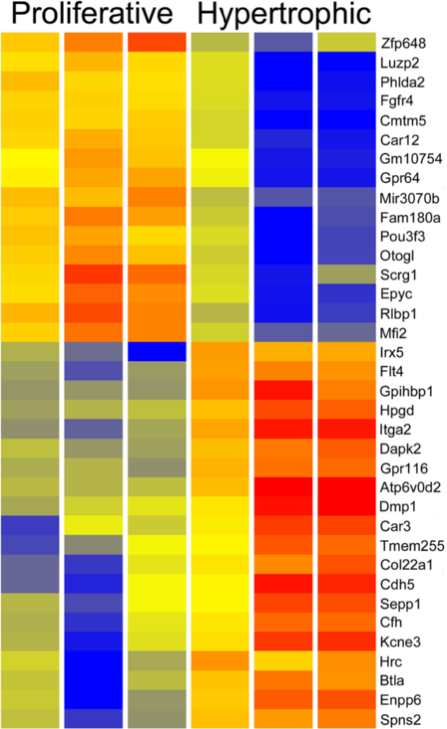
Heatmap comparing wild type proliferative and hypertrophic compartments. The 36 genes with the greatest fold change (≥10) are shown. The RNA-Seq results define the changing gene expression patterns, including growth factors, receptors and transcription factors, as cells enter the hypertrophic phase. Red reflects strong, blue represents weak, and yellow indicates intermediate expression

The resulting RNA-Seq data defines the WT changing gene expression programs as cells progress from the resting to proliferative and hypertrophic phases of normal bone development. In comparing WT resting and proliferative compartments 347 genes with differential expression were identified (*P* ≤ 0.05, FC ≥ 2) (Additional file 1: Table S1). A heatmap of 40 genes with FC ≥ 5 is shown in Fig. 5. ToppGene was used to define the distinct molecular functions and biological processes of the resting and proliferative zones (Additional file 2: Table S2). A similar comparison of the WT hypertrophic versus WT proliferative compartments revealed 638 genes with FC ≥ 2 (Additional file 3: Table S3). The 36 genes with FC ≥ 10 are shown in the heatmap of Fig. 6. Functional enrichment analysis revealed increased expression in the hypertrophic compartment of genes involved in cell adhesion, signaling, cell migration and vasculature development (Additional file 4: Table S4).

The LCM/RNA-Seq data described in this report provides, to our knowledge, the first RNA-Seq definition of the changing gene expression programs driving the resting, proliferative and hypertrophic compartments of WT endochondral bone development. The results identify a large number of previously defined gene expression differences, thereby providing an historic validation of the dataset. For examples, up-regulated genes in the proliferating compartment compared to resting included *Ihh* [47], *Panx3* [48], *Dlx5* [49], and *Sp7* [50]. The RNA-Seq identified genes with strongest expression in the hypertrophic compartment, again in agreement with previous studies, included *Col10a1* [50], *Flt4* (Vegf-R3) [51], and *Dmp1* [52]. The LCM/RNA-Seq *dataset* described in this report adds to previous microarray studies of gene expression patterns in the developing bone [53, 54].

**Fig. 7:**
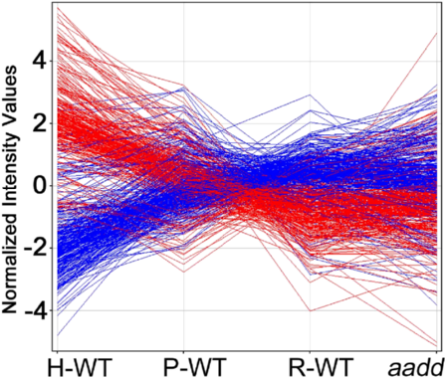
Series graph comparison of wild type and *aadd* Hox mutant gene expression profiles. The 547 genes with fold change ≥ 5 in any pairwise comparison are included. Each line represents a gene. H-WT, hypertrophic wild type, P-WT, proliferative wild type, R-WT, resting wild type, and *aadd*, double homozygous mutant for *Hoxa9,10,11* and *Hoxd9,10,11*, are shown. The hypertrophic compartment shows the most divergent gene expression pattern. The *aadd* mutant most closely resembles the resting wild type compartment of the forming bone, consistent with the histologic comparison

ANOVA of all compartments, including both WT and *aadd*, identified 547 genes with FC ≥ 5 in any pairwise comparison. A series graph shows the gene expression relationships of compartments (Fig. 7). The hypertrophic zone shows the most divergent gene expression pattern, and the *aadd* mutant cells are clearly most similar to the resting cells of the WT, consistent with their histologic appearance. The proliferative cells appear intermediate in gene expression profile compared to resting and hypertrophic.

### Wild type versus *aadd* mutant

**Fig. 8:**
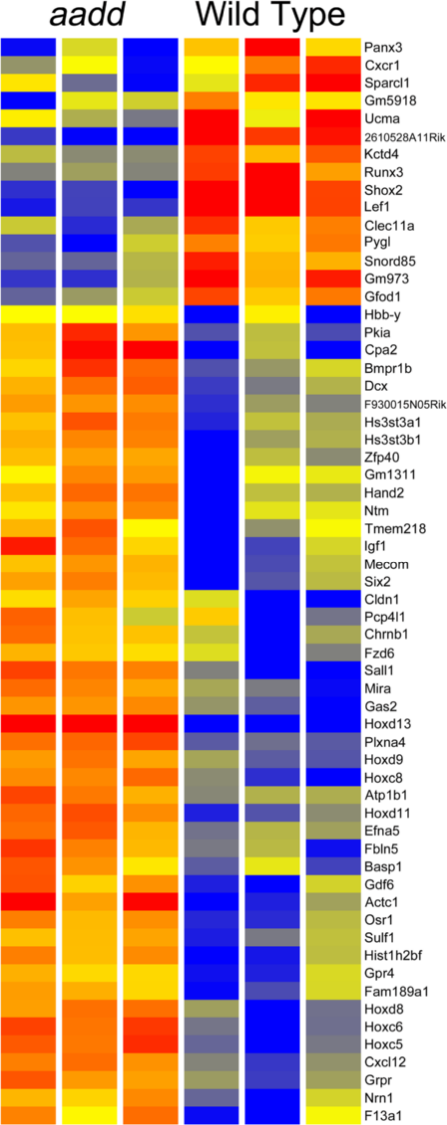
Heatmap comparison of Hox mutant and resting wild type gene expression patterns. The 61 genes with the greatest fold change (≥3) are shown. The most strongly down-regulated genes in *aadd* (*Hoxa9,10,11* ^−/−^;*Hoxd9,10,11* ^−/−^) mutants include *Lef1*, *Shox2* and *Runx3*. The most strongly up-regulated genes include *Sall1*, *Six2*, *Gas1*, *Gas2* and *Osr1*

We compared the gene expression patterns of the *aadd* mutant and WT resting cells to better understand the nature of the mutant defect in progression to the proliferative phase. Because of the overall similarity of mutant and WT resting compartments we reduced the stringency of the screen to *P* ≤ 0.05, FC ≥ 1.5, to find as many differences as possible. This identified 845 genes with differential expression (Additional file 5: Table S5). Functional enrichment analysis identified molecular functions and biological processes and their associated genes (Additional file 6: Table S6). A more stringent screen of the data, requiring FC ≥ 3, gave 61 genes (Fig. 8).

### Genes with reduced expression in *aadd* mutant limbs

The LCM/RNA-Seq data identified a number of known critical regulators of limb development that were strongly down regulated in the *aadd* mutants. The short stature homeobox gene *Shox2* was reduced in expression by about ten fold. Interestingly, *Shox* mutant phenotypes are characterized by mesomelia, with disproportionate shortening of zeugopods [55]. Another transcription factor gene, *Runx3*, showed about a five fold reduction in *aadd* mutants. *Runx3* mutants show normal skeletal development, but double *Runx2/3* mutants have a complete failure of chondrocyte maturation [56], much more severe than observed for mice with only mutant *Runx2.* There is an interesting positive regulatory loop between *Shox2* and *Runx2/3*. In *Shox2* mutants both *Runx2* and *Runx3* show reduced expression [57, 58]. Conversely, *Runx2*^−/−^/*Runx3*^−/−^ mutants show reduced expression of *Shox2* [35]*.*

Hox regulation of Shox and Runx genes has been previously described. *Hoxa11*^−/−^/*Hoxd11*^−/−^ mutants show reduced expression of both *Shox2* and *Runx2* [35] and *Hoxd13* directly targets *Runx2* [59]. Of interest we observed strong down regulation of *Runx3* in *aadd* mutants, but no change in the expression *Runx2.* The reasons for the apparent discrepancy are unclear, but could relate to the distinct Hox mutations studied (*aadd*, *Hoxa11*^−/−^/*Hoxd11*^−/−^, *Hoxd13*^spdh/+^). In any event the combined results of multiple studies now place Hox genes upstream of the of the key *Shox2*;*Runx2/3* regulators of limb development.

Several genes important in growth factor signaling also showed reduced expression in *aadd* mutants. These included the insulin receptor *Irs1* gene, which is required for IGF1 and insulin signaling. *Isr1*^−/−^ mice have shortened limbs with reduced proliferative and hypertrophic zones, resembling the *aadd* mutants [60]. The *Bmp7* gene was also down-regulated. Bmp7 induces new bone formation and stimulates osteoblast proliferation and differentiation [61]. Reduced *Bmp7* expression was also previously reported in *Hoxa13* mutants [62].

*Lef1*, encoding a transcription factor effector of Wnt signaling, was reduced about ten fold in expression in *aadd* mutants. Canonical Wnt signaling promotes chondrocyte and osteoblast differentiation [63, 64]. Loss of Wnt signaling in the developing bone delays chondrocyte hypertrophy, and gives a shorter hypertrophic zone [58]. *Gli1* was also down regulated in mutants. *Gli1* encodes a transcription factor mediating Hedgehog signaling, which is essential for osteoblast formation [47].

We observed no change in the expression levels of *Bmp2* or *Sox9* in *aadd* mutant LCM samples. This is of interest because *Hoxa13* mutants show reduced expression of *Bmp2* in the autopod [62], and evidence strongly supporting Hox regulation of *Sox9 h*as been previously reported [65]. These results provide examples of Hox gene and tissue context specificity.

### Genes up-regulated in *aadd* mutant limbs

There were also a number of interesting strongly up-regulated genes in *aadd* mutants. Several Hox genes showed up-regulation, perhaps reflecting compensatory expression. We also observed a dramatic up-regulation of the homeobox transcription factor gene *Six2* in the *aadd* mutants. *Six2* expression has been shown to prevent maturation to hypertrophic chondrocytes, and to promote chondrocyte proliferation [66]. Hox repression of *Six2* expression was previously reported in branchial arch development. In particular, *Six2* was shown to be a direct downstream target of *Hoxa2*, and ectopic expression of *Six2* in the mutant was shown to directly contribute to the mutant phenotype [67]. In contrast with what we observe in the developing limbs, in the kidney Hox11 proteins interact with Pax2 and Eya1 to drive Six2 expression [68, 69]. Activation of *Six2* by Hox11 has been shown to require domains both N- and C-terminal to the homeodomain [70].

Several additional transcription factors of known importance in limb development were up-regulated in *aadd* mutant limbs. The homeobox *Pknox2* gene was elevated in expression about three fold. Transgenic overexpression of *Pknox2* in the developing limb causes dramatic shortening of the zeugopod, with chondrocyte differentiation blocked at an early stage, similar to the *aadd* phenotype [71].

The *aadd* mutants also showed up-regulation of *Zfp467,* which suppresses osteoblast differentiation [72]. In addition, *Tbx18* was up-regulated in *aadd* mutants, and *Tbx18* mutants have shortened limbs [73]. The *Sall1* gene also showed elevated expression in mutants. *Sall1* mutation has been associated with Townes-Brocks syndrome, which can include hand and foot abnormalities, with hypoplastic thumbs, syndactyly, and fusion of wrist bones. We also observed up-regulation of *Hand2*, which is an inhibitor of Runx and inhibits osteoblast differentiation [74].

Several genes associated with joints were over expressed in the *aadd* mutants. *Osr1* was up-regulated four fold in mutants. *Osr1* expression has been linked to reduced chondrogenesis [75]. *Osr1* and *Osr2* are normally expressed in joint forming regions [76]. Of interest, *Osr1* and *Six2*, both strongly up-regulated in the *aadd* mutants, have been shown to synergistically interact to maintain progenitor cells during kidney development [77]. *Osr1*^*−/−*^/*Osr2*^*−/−*^ double mutants show fusions of bones with absent joints, and loss of *Gdf5* expression, another marker of joint formation [76]. In the *aadd* mutants *Gdf5* expression was elevated, consistent with its positive regulation by *Osr1. Gdf5* is a member of the BMP family, and mutations result in reduced bone length and perturbed joint formation [78]. *Dcx*, normally expressed in articular chondrocytes at joints, was also strongly up-regulated [79]. Of interest, however, several other joint markers, including *Osr2, Cux1,* and *Erg,* did not show elevated expression.

In addition to *Gdf5* two other genes involved in BMP signaling were up-regulated in mutants, including the BMP receptor encoding gene *Bmpr1b*, which is required for osteogenesis *in vitro* [80]. Of interest, the transgenic overexpression of the closely related *Bmpr1a* causes shortening of long bones [81]. *Sulf1* was also overexpressed in *aadd* mutants. *Sulf1* and *Sulf2* are functionally redundant and double mutants show a short stature phenotype [82]. Sulf1 and Sulf2 are involved in the synthesis of cell surface heparin sulfate required for the binding of the Noggin antagonist of BMP signaling [83].

The growth arrest specific genes *Gas1* and *Gas2* were also up-regulated in the mutant limbs. Gas1 is a positive component of the SHH signaling pathway and *Gas1* mutants show bone malformations [84]. *Gas2* expression is highly correlated with apoptosis [85], consistent with the elevated apoptosis previously observed in *Hoxa11*^−/−^/*Hoxd11*^−/−^ limbs [34].

Other genes of particular interest that showed elevated expression in the mutant limbs included *Kcnrg*, which encodes a regulator of potassium channels. *Kcnrg* expression is anti-proliferative and pro-apoptotic [86]. *Dkk3*, yet another osteoblast antagonist [87], was also up-regulated. *Igf1*, a positive regulator of bone growth, also showed increased expression in mutants. Global deletion of *Igf1* results in dwarfism [88, 89] and tissue specific Cre mediated deletion in chondrocytes also gives reduced bone length [90].

It is interesting to note that up-regulation of *Igf1,* as well as *Tbx18, Gdf5* and *Sulf1,* might be expected to increase bone length, and not decrease it, since mutations in these genes cause shortened bones. This elevated expression could represent a compensatory response, with the developing bone attempting to recover growth lost through other perturbed pathways. Alternatively, in some cases during development correct gene expression level is critically important, with either over or under expression giving a similar phenotype.

### RNA-Seq validations

**Fig. 9:**
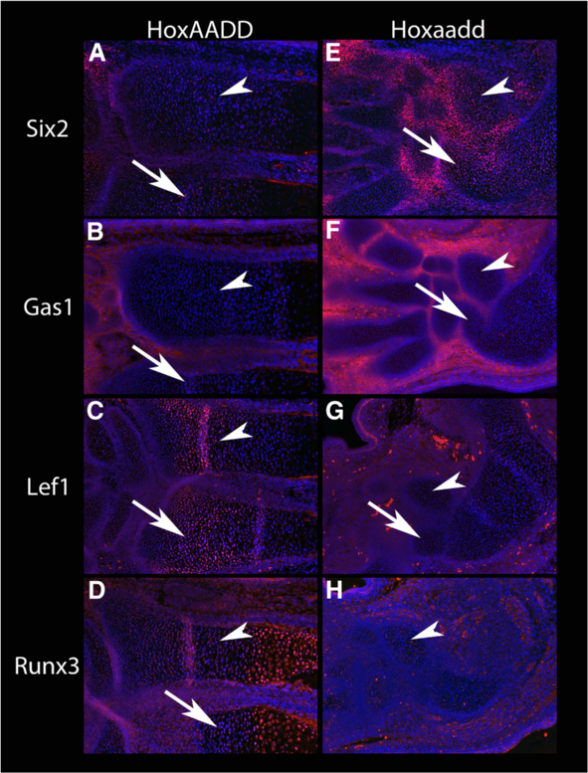
Immunofluorescent staining of E15.5 Wild type HoxAADD (**a**–**d**) and mutant Hoxaadd (*Hoxa9,10,11* ^−/−^;*Hoxd9,10,11* ^−/−^) (**e**–**h**) forelimbs. Arrowhead: radius, Arrow: ulna, the autopod is oriented to the left of the image. **a** and **e**: Six2 immunostaining, showing an increased expression in mutant chondrocytes. **b** and **f**: Gas1 staining, showing an increase in mutant limbs that is restricted to cells flanking chondrocytes, consistent with the inclusion of some perichondrial cells in the LCM samples. **c** and **g**: Lef1 staining, showing an absence of staining in mutant chondrocytes. **d** and **h**: Runx3 staining, showing an absence of staining in mutant chondrocytes

We used immunohistochemistry to validate RNA-Seq predicted gene expression differences. Genes were selected for validations based on high expression level and strong fold change. Immunostains confirmed the elevated expression in *aadd* mutants of both Six2 and Gas1. Six2 showed elevated expression that was strongest between the chondrogenic zones but included the forming ulna and radius as well (Fig. 9a–d). Gas1 also showed substantial up-regulation that was primarily restricted to the interchondrogenic regions. In addition, Lef1 and Runx3 showed reduced expression in the mutant ulna and radius (Fig. 9e–h). Some of the detected difference likely reflect the distinct cell types present.

## Conclusions

In summary, we have studied Hox function in the developing limbs using mice with frameshift mutations in the first exons of multiple flanking Hox genes. These mice provide precise genetic tools with disrupted coding function, while shared regional enhancers remain intact, thereby minimizing effects on the expression of remaining Hox genes.

The *aadd* mutants, with twelve mutated flanking and paralogous Hox alleles, show a very significant reduction of *Shh* expression in the ZPA. The effect is similar to that seen in mice with entire deletions of the HoxA and HoxD clusters, and much more severe than observed in mice with only *Hoxa11*^−/−^/*Hoxd11*^−/−^ mutations. This shows that within the HoxA and HoxD clusters it is the 9,10,11 paralog genes that are primarily responsible for driving *Shh* expression. The LCM/RNA-Seq analysis defines the changing gene expression programs in wild type limbs as cells progress from resting to proliferative and hypertrophic phases. The *aadd* mutants showed altered expression of a number of known key regulators of endochondral bone formation. Several of the up-regulated genes are known to inhibit chondrocyte or osteoblast differentiation, including *Pknox2*, *Zfp467*, *Hand2* and *Dkk3*. Other up-regulated genes are involved in joint formation (*Osr1*, *Gdf5*, *Dcx*, *Sall1*), BMP signaling (*Gdf5*, *Sulf1*, *Bmpr1b*), and growth arrest, apoptosis and reduced proliferation (*Gas1*, *Gas2*, *Kcnrg*). The altered expression of these genes defines key perturbed pathways downstream of *Hoxa9,10,11*/*Hoxd9,10,11* in the developing limb zeugopod.

## Additional files

Additional file 1: Table S1. Proliferative versus resting gene expression differences. Additional file 2: Table S2. Functional enrichment analysis of proliferative versus resting. Additional file 3: Table S3. Hypertrophic versus proliferative gene expression differences. Additional file 4: Table S4. Functional enrichment analysis of hypertrophic versus proliferative. Additional file 5: Table S5. aadd mutant versus resting wild type gene expression differences. Additional file 6: Table S6. Functional enrichment analysis of aadd mutant versus resting wild type.

## Abbreviations

r: Rhombomere
AER: Apical ectodermal ridge
Shh: Sonic hedgehog
FGF: Fibroblast growth factor
ZPA: Zone of polarizing activity
LCM: Laser capture microdissection
*aadd*: *Hoxa9,10,11*^−/−^/*Hoxd9,10,11*^−/−^
*AADD*: *Hoxa9,10,11*^+/+^/*Hoxd9,10,11*^+/+^
